# An Extremely Rare Case of Squamous Cell Carcinoma Following Treatment for External Auditory Canal Papilloma

**DOI:** 10.7759/cureus.84755

**Published:** 2025-05-24

**Authors:** Yumi Ohta, Yoshiyuki Ozono, Motoyuki Suzuki, Takashi Sato, Hidenori Inohara

**Affiliations:** 1 Department of Otorhinolaryngology-Head and Neck Surgery, National Hospital Organization Osaka Medical Center, Osaka, JPN; 2 Department of Otorhinolaryngology-Head and Neck Surgery, The University of Osaka Faculty of Medicine, Suita, JPN

**Keywords:** external auditory canal, human papilloma virus, radiation-induced cancer, squamous cell carcinoma, squamous papilloma

## Abstract

The case is of a 60-year-old woman with a history of radiotherapy for parotid adenoid cystic carcinoma. Twenty years after irradiation, a papilloma developed in the left external auditory canal. Nine years after excision of the papilloma, a squamous cell carcinoma developed in the same region. This is a rare case of squamous cell carcinoma following treatment for a papilloma and is considered radiation-induced cancer. Because re-irradiation is not possible as a treatment for radiation-induced cancer, it is necessary to diagnose the cancer early so that curative resection is possible. Multiple biopsies should be considered when malignancy is suspected because a single biopsy may not be sufficient to diagnose an auditory canal tumor.

## Introduction

In the head and neck region, papillomas of the nasal sinuses, pharynx, and larynx are relatively common, but papillomas of the external auditory canal are rare [1,2]. Most reports of external auditory canal papillomas are from Taiwan and southern China [1,2], but there are few reports from Japan. The incidence of external auditory canal malignancies is 1-6 per million population [3-6], or less than 0.2% of all head and neck cancers [4,5], which are also rare. Although the incidence of external auditory canal malignancy is low, it is known to occur following radiation to other parts of the head and neck. There have been reports of squamous cell carcinoma in the temporal bone region after radiation for nasopharyngeal carcinoma [7-9]. The reported incidence of radiation-induced tumors ranges from 0.04% to 7% in post-radiation nasopharyngeal carcinoma [7]. This suggests that radiation-induced cancer may occur when the external auditory canal is included in the radiation field. As far as we know, there is only one reported case of a papilloma in the external auditory canal that subsequently developed into a squamous cell carcinoma [10]. Here, we report an extremely rare case of squamous cell carcinoma that developed after the treatment of a papilloma of the external auditory canal. Although a period of papilloma in the process of cancer development is extremely rare, this case is thought to be a radiation-induced cancer.

## Case presentation

Subject and medical history

**Figure 1:**
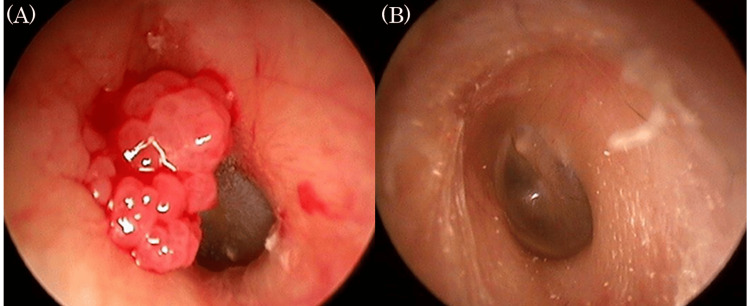
Endoscopic findings in the left external auditory canal (A) On initial examination, a papillary mass was found, with its base in the anterior wall of the external auditory canal. (B) Two years after resection, there were no abnormal findings.

The patient was a 60-year-old woman at the time of the initial examination. She had been referred by her family doctor for a mass in her left external auditory canal. On initial examination, a papillary mass was found in the left external auditory canal, with its base in the anterior wall of the external auditory canal (Figure 1A).

After an outpatient biopsy, the pathological diagnosis was papilloma. The tumor was therefore removed as a radical operation by resection of the base with a CO2 laser (power 10 W, diameter 0.3 mm, continuous irradiation mode).

The patient had diabetes and rheumatoid arthritis but was well-controlled with oral medication. The patient had a medical history of surgery and irradiation (50 Gy/25 fr) for cancer of the left parotid gland (adenoid cystic carcinoma) at age 40 (20 years before the initial diagnosis). The treatment for parotid gland cancer was surgical removal of the tumor, followed by radiation to the entire neck. The left external auditory canal was also included in the radiation field.

Pathological findings (squamous papilloma)

**Figure 2:**
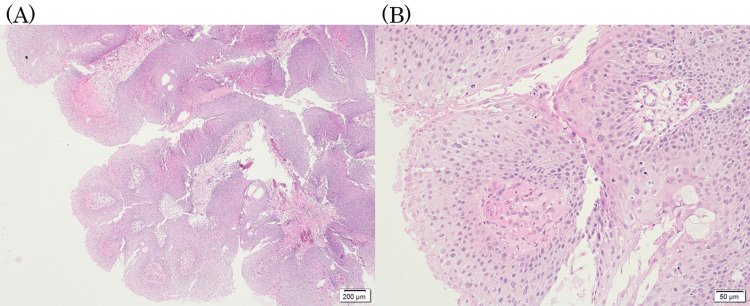
Papilloma pathological findings. The papillary tissue was covered by a stratified squamous epithelium, with a neutrophilic infiltrate in the stroma and epithelium. (A) ×40 and (B) ×200 hematoxylin and eosin staining.

The papillary tissue was covered by a stratified squamous epithelium, with a neutrophilic infiltrate in the stroma and epithelium. The nuclei of the epithelial cells were slightly enlarged, with a prominent nuclear cleavage pattern down to the middle layers, but the stratified differentiation of the epithelium was preserved. The diagnosis was squamous papilloma with reactive changes and atypia (Figure 2).

 Human papillomavirus analysis

The TaKaRa PCR Human Papillomavirus Typing Set (Takara Bio Inc. Kusatsu, Japan), which can test for types 6, 11, 16, 18, 31, 33, 35, 52b, and 58, and the nested PCR method (types 16, 18, 31, 33, 35, 45, 52 and 58) were used, using the method proposed by Haws [11]. No HPV of any type was detected.

Findings and treatment nine years after initial treatment

The patient was followed for two years postoperatively, and follow-up was discontinued, as there was no recurrence of papilloma (Figure 1B). Seven years later, she was referred back by her family doctor for recurrent otitis externa that had not healed.

**Figure 3:**
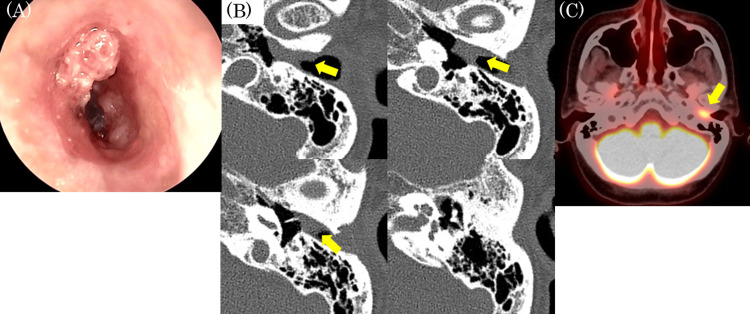
Findings at the time of re-referral (A) Endoscopic findings in the left external auditory canal: Papillary lesions were found around the external auditory canal and on the posterior half of the tympanic membrane. (B) CT (axial): The soft tissue shadow was localized within the left auditory canal and there was no obvious bone destruction. (C) PET-CT: Abnormal accumulation was found only in the left auditory canal.

**Figure 4:**
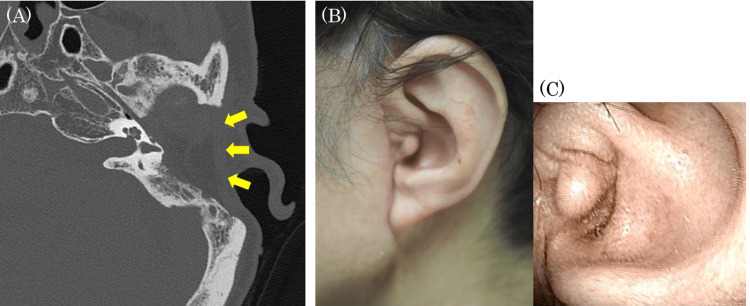
Postoperative findings (A) CT image six months after surgery: filled with a pedicled temporal fascia and abdominal fat; (B) Photograph of the external ear (whole auricle); (C) Photograph of the external ear (closed portion of the external auditory canal)

At the time of re-referral, papillary lesions were found around the entire circumference of the left external auditory canal, as well as on the posterior half of the tympanic membrane (Figure 3A). An outpatient biopsy revealed a papilloma with severe dysplasia, but malignancy could not be ruled out, so a further excisional biopsy was performed under general anesthesia. Even if it was a benign papilloma rather than a malignant tumor, there was a risk of seeding the tumor in the tympanic cavity if it entered the cavity during the surgical procedure; therefore, the area of the tympanic membrane was not excised and the lesion was excised from the anterior wall of the external auditory canal to the inferior wall and sent for pathological examination. The pathological diagnosis was squamous cell carcinoma. CT showed no obvious bone destruction, and soft tissue shadows were localized within the ear canal (Figure 3B), and positron emission tomography-computed tomography (PET-CT) showed abnormal accumulation only in the left ear canal (Figure 3C), leading to the diagnosis of cT1N0M0 according to the modified Pittsburgh classification. One month after the biopsy under general anesthesia, a lateral temporal bone resection and filling with a pedicled temporal fascia and abdominal fat was performed. The external auditory canal was closed using a method called the blind sac technique. The postoperative course was uneventful, and local recurrence or metastases were observed six months postoperatively. Figure 4 shows a postoperative CT image and external ear photographs.

Pathological findings (squamous cell carcinoma)

**Figure 5:**
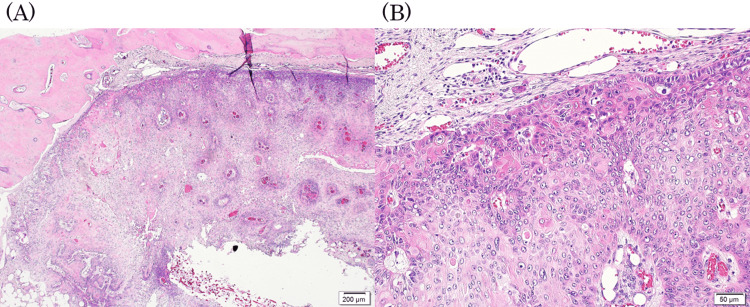
Squamous cell carcinoma pathological findings Atypical multilayered squamous epithelium with darkly stained and enlarged round or oval nuclei and eosinophilic reticulum was seen in many parts of the lesion, with full-layered growth within the epithelium. (A) ×40 and (B) ×200 hematoxylin and eosin staining.

Atypical multilayered squamous epithelium with darkly stained and enlarged round or oval nuclei and eosinophilic reticulum was seen in many parts of the lesion, with full-layered growth within the epithelium. The diagnosis was squamous cell carcinoma. Slight infiltration of the subepithelial stroma was observed in a very small area. There was no evidence of invasion or destruction of bone tissue, no vascular or perineural invasion, and the tumor had negative margins (Figure 5).

## Discussion

There is only one case report of a papilloma in the external auditory canal that subsequently developed into squamous cell carcinoma [7]. The report stated that the squamous cell carcinoma appeared to have arisen from the background papillomatous, hyperkeratotic squamous epithelium. This is the same mechanism by which squamous cell carcinoma occurs in nasal sinus papilloma. We do not believe that the present case follows the same mechanism.

If a tumor has been treated in the past and a new tumor arises close to the treated site, the new tumor could be either a recurrence of the primary tumor or a secondary cancer caused by the previous cancer treatment. Radiation-induced cancer is defined as a malignant tumor that occurs within a previous radiation field, has histologic features different from the primary disease, and occurs at least five years after radiotherapy [4,12]. In the present case, the parotid carcinoma was an adenoid cystic carcinoma, and the external ear carcinoma was a squamous cell carcinoma, i.e., different histological types and sites of origin. Therefore, radiation-induced cancer was suspected. This case had a history of papillomas that preceded the development of the malignant tumor. Possible factors in the papilloma’s development include radiation-induced epithelial damage and impaired local immune function. Diabetes and rheumatoid arthritis, which require treatment, may also be a factor in reduced local immune function.

HPV is implicated in the development of papillomas. PCR subtyping analysis for HPV was performed in this case, but HPV was not detected. In the head and neck region, papillomas are relatively more common in the nasal cavity and sinuses, but HPV detection rates are not high in these areas [13]. Therefore, even if HPV is not detected, it cannot be ruled out that HPV is involved in tumor development. HPV is a contact infection; it does not infect healthy skin but infects epithelial stem cells from slightly damaged areas of skin or mucous membranes. It has been speculated that papillomas of the external auditory canal may be caused by infection from the insertion of HPV-contaminated fingers or earpicks into the external auditory canal [1].

In papillomas, it is likely that HPV subclinically infects cells surrounding the tumor, and the surrounding normal tissue should also be excised. Electrocautery, CO2 laser ablation, or cryotherapy is recommended [1]. In this case, CO2 laser ablation was performed, and the patient was followed for two years. However, the lesion recurred in the external auditory canal. Although there is no clear standard for the length of follow-up after papilloma resection, Luo reported a 22% recurrence rate of external auditory canal papillomas and mentioned the need for long-term follow-up [2]. In this patient, a papilloma developed in the previously irradiated field, requiring more careful long-term follow-up.

In addition to surgery and chemotherapy, radiotherapy plays a central role in the treatment of malignant tumors of the head and neck. Radiotherapy inevitably causes damage to the surrounding normal tissue. The temporal bone is particularly affected by radiation for nasopharyngeal, oropharyngeal, parotid, and brain tumors. Effects of including the temporal bone in the irradiated area include the risk of sensorineural hearing loss, Eustachian tube dysfunction, osteonecrosis, otitis externa, and malignancy [12]. If the irradiated area includes the temporal bone, the patient should be monitored for these signs.

The only treatment option for radiation-induced cancer is surgical resection and chemotherapy; re-irradiation is not an option owing to the risk of tissue necrosis [4,12]. Therefore, it is important to detect the disease at an early stage when radical resection is possible. However, external auditory canal malignancies may not be diagnosed with a single histologic examination. Some squamous cell carcinomas have a papillary morphology even in cases without a history of papilloma, as in the present case. The diagnosis should not be based on a single biopsy alone, but on a combination of disease history and imaging findings, and in some cases, multiple biopsies should be performed to make an accurate diagnosis.

How long a patient should be followed after cancer treatment is not clearly defined. It has been reported that the average time from the first cancer treatment to the development of radiation-induced cancer is 13.4 years [12], and it is not feasible to follow the patient in a medical institution for that long. In general, follow-up after treatment for head and neck cancer often ends after five years. However, because radiation-induced cancers occur over a long period of time, it is advisable to educate patients who have undergone radiation therapy to remain vigilant even after five years. It may be necessary to inform patients and instruct them to seek medical attention if symptoms occur.

## Conclusions

We report an extremely rare case in which a papilloma of the external auditory canal developed 20 years after radiotherapy for parotid adenoid cystic carcinoma, followed by squamous cell carcinoma 9 years later. It was considered radiation-induced cancer. A biopsy was performed for early diagnosis, and radical surgery was performed. The possibility of radiation-induced cancer should be considered if there is a history of radiation therapy.
